# Improved DEMATEL-ISM integration approach for complex systems

**DOI:** 10.1371/journal.pone.0254694

**Published:** 2021-07-16

**Authors:** Jih-Kuang Chen

**Affiliations:** Economics and Management College, Zhaoqing University, Zhaoqing, China; University of Defence in Belgrade, SERBIA

## Abstract

**Purpose:**

Decision Making Trial and Evaluation Laboratory (DEMATEL) and Interpretive Structural Modeling (ISM) are commonly used separately, but also may be combined per their common characteristics to identify causal relationships and hierarchical structure among factors in complex systems with a relatively small computational burden. The purpose of this study is to establish an improved DEMATEL-ISM integration approach to remedy the disadvantages of the traditional DEMATEL-ISM integration method. A case study was conducted to compare the proposed improved integration approach against the traditional integration method, and to validate its feasibility and effectiveness.

**Methods:**

The proposed improved DEMATEL-ISM integration approach has two main parts: a threshold determination via maximum mean de-entropy (MMDE) method and an additional transitivity check process. The factors influencing China’s rural-urban floating population’s willingness to participate in social insurance was analyzed as a case study.

**Results:**

The traditional and improved methods show notable differences in the hierarchical factor structure and the inner influence relationship among factors that they respectively reveal. The traditional integration approach results in some irrationality, while the improved approach does not.

**Originality:**

This study confirms the importance of proper threshold determination and reachability matrix transitivity checking during DEMATEL-ISM integration. The improved approach includes a scientific threshold determination method based on the MMDE method, plus a transitivity check of the reachability matrix with necessary corrections to ensure its soundness. It can be straightforwardly operated at a relatively low computational burden while providing accurate analysis results.

## Introduction

Modern organizations are faced with increasingly intense complexity and uncertainty in today’s competitive market environment. Organizational decision-makers and managers must quickly and correctly grasp a complicated array of contradictions to maintain economic competitiveness. Decision Making Trial and Evaluation Laboratory (DEMATEL) is an analytical method that was proposed to facilitate decision-making across complex factors [1]. DEMATEL serves to first simplify the multi-factor system structure and then screen out the main influence factors. Based on graph theory, DEMATEL supports the development of knowledge and experience; it allows for analysis of the logical correlations and direct-influence relationships between various factors in complex systems, thus revealing key factors. Due to the universality and simplicity of its mechanism, DEMATEL has garnered a great deal of scholarly interest in recent years across engineering, management science, supply chain management, and other fields [2–4]. Interpretive Structural Modeling (ISM) was established to decompose a complex system into several smaller systems, then build a structured model of the complex system [5]. ISM has multi-level hierarchy and can express ambiguous systems with intuitive structural relations, facilitating the objective analysis of complex problems. It has also been extensively investigated by researchers in multiple fields [6–8].

Many researchers have used DEMATEL and ISM separately to identify main influence factors and to construct multi-level hierarchical structured models of them, thus obtaining complete information [9–11]. However, separate calculation processes are overly complex and have an excessive calculation burden. DEMATEL and ISM methods both express expert evaluation information via relation matrix and reflect the influence relationships between factors. Zhao, Zhang, and Li (2006) [12] proposed a DEMATEL-ISM integration approach. They argue that as the total-relation matrix of DEMATEL contains more information than the reachability matrix of ISM, and the reachability matrix is more difficult to calculate, that the hierarchical structure of the complex system can be divided by the total-relation matrix of DEMATEL and then transformed into an ISM reachability matrix. However, this approach (from here on referred to as the “traditional integration” method or approach) has notable drawbacks and may lead to deviation in the ISM analysis results and drive down the effectiveness of any countermeasures implemented. (Details regarding its drawbacks and solutions are given in Section 3.) This traditional DEMATEL-ISM integration method has been adopted in many previous studies (Section 2). The purpose of the present study is to establish an improved DEMATEL-ISM integration approach to remedy the disadvantages of the traditional integration method.

The study of the factors influencing China’s rural-urban floating population’s willingness to participate in social insurance was analyzed by the improved integration approach versus the traditional integration method to validate its feasibility and effectiveness. The factors influencing social insurance willingness of the rural-urban floating population in the Guangdong-Hong Kong-Macao Greater Bay Area was taken as an example to test the improved integration approach by comparison against the traditional integration method. Differences emerged between the two approaches, including the hierarchical structure and inner influence relationships they respectively reveal. The results of this work may serve as a helpful reference for further research on the case study topic.

## Literature review

### Step-wise traditional DEMATEL-ISM integration method

To operate the traditional DEMATEL-ISM integration method, DEMATEL is first used to determine the direct relation level between two factors through expert evaluation (typically on a 0–4 scale) to form a direct-relation matrix X:

$$X=[\begin{array}{cccc} 0 & x_{12} & \ldots & x_{1n}\\ x_{21} & 0 & \ldots & x_{2n}\\ \vdots & \vdots & 0 & \vdots \\ x_{n1} & x_{n2} & \ldots & 0 \end{array}]$$ (1)

After taking the maximum value of row sum as the normalized cardinality, the transformation matrix X becomes the normalized direct-relation matrix N:

$$N=\frac{1}{\underset{0\leq i\leq n}{\max}\sum_{j=1}^{n}x_{ij}}$$ (2)

The total-relation matrix T is then calculated as follows:

$$T=N{\left(I-N\right)}^{-1}$$ (3)

where I is the identity matrix and ^-1^ is the inverse matrix.

The influence level and the relationship level of each factor can be added to determine “prominence”, while the influence level and the relationship level of the system factor can be subtracted to determine “relation”. The prominence and relation of the factors form a two-dimensional coordinate system. The positions of each factor in the coordinate system are marked, the importance of each factor is analyzed, and suggestions are put forward for the system.

In the traditional integration approach, the total-relation matrix T does not account for the influence of a factor on itself, so the identity matrix I is added to form the matrix H (H = T+I). The value range of elements in the ISM’s reachability matrix is {0,1}, so experts or decision-makers can scientifically and reasonably set the threshold λ to suit the problem at hand. The matrix H can be directly converted into the reachability matrix K after comparison based on the following equation:

$$k_{ij}=\{\begin{array}{c} 1,h_{ij}\geq \lambda \\ 0,h_{ij}<\lambda \end{array}$$ (4)

The hierarchical structure of system factors can then be established based on the reachability set and antecedent set provided by the ISM reachability matrix. The steps of this process are as follows.

1. Determine the reachability set and antecedent set of each factor
  The reachability set, a set of factors corresponding to all rows with 1 element in the i-th row of the reachability matrix, is represented by R(i). The antecedent set, a set of factors corresponding to all columns with 1 element in the i-th column of the reachability matrix, is represented by Q(i).
2. Identify the intersection set C(i)
  C(i) is the intersection set of the reachability set R(i) and antecedent set Q(i). When C(i) = R(i) is satisfied, the corresponding factor is the first-level factor and i-th row and i-th column can be delimited from the reachability matrix K. This step is repeated until all factors are crossed out. A multi-level hierarchical structure of factors can then be established according to the order in which the factors are crossed out. This technique saves a great deal of complicated matrix calculations and is not particularly prone to user error.

### Previous applications of traditional DEMATEL-ISM integration method

The traditional DEMATEL-ISM integration method has been adopted by many previous researchers. Shen, Sun, Zhang et al. (2014) [13], for example, used it to build a logical relationship of many failure subsystems; failure mode effects and criticality analysis (FMECA) was then applied qualitative analysis of the direction of reliability improvement. Hou and Xiao (2015) [14] used it to identify the critical success factors (CSFs) that impact the linkage mechanism between government and non-profit organizations tasked with geo-disaster emergency decisions. Zhang and Luo (2017) [15] used it to analyze the influencing factors of runway incursions and their mechanisms, offering a novel decision-making idea for runway incursion control. Shen, Li, Shi et al. (2018) [16] used it to construct a multi-level hierarchical structure of influencing factors on the economics of a distributed natural gas combined cooling, heating, and power (DNG-CCHP) system.

There have been many other valuable contributions to the literature. Wang, Cao, and Zhou (2018) [17] used traditional DEMATEL-ISM integration to establish a hierarchical model of influencing factors and mechanisms related to mining safety. Xie and Liu (2019) [18] used it to establish a hierarchical structure of influencing factors and to distinguish cause and effect factors. Li, Wang, Dubljevic et al. (2019) [19] used it to establish a hierarchical network model, which they mapped to a Bayesian network (BN) to further transform the conditional probability distribution; they quantified the strength of coupling relationships in an accident-causing system accordingly to determine main paths resulting in system failure. Sun, Zhou, Zhang et al. (2019) [20] used it to integrate the interaction direction and intensity between failure factors and hierarchical factor structures, then used the analytic network process (ANP) to determine the relative importance of CNC equipment fault factors.

Shakeria and Khalilzadeh (2020) [21] used this traditional method to identify and determine sequences and relationship factors affecting project communications and their clusters to inform organizational and project managers in the context of project communications. Cui, Zhu, and Wand (2020) [22] used it to explore the influencing factors of collaborative innovation in low-carbon technology in China in the context of Paris Agreement goals. Yue and Han (2020) [23] used it to determine the causative attributes of Chinese airline company safety by creating an indicator system for safety risk factors. Liu, Dou, Yang (2021) [24] used it to analyze the influencing factors of cross-border e-commerce supply chain resilience (CBSCR), so as to further enhance the competitiveness of a global supply chain.

## Drawbacks and potential solutions of traditional integration method

As mentioned above, it merits consideration whether the total-relation matrix T takes into account the influence of a given factor on itself. The total-relation matrix T is derived from $\underset{t->\infty }{\lim}\left(X+X^{2}+\cdots +X^{t}\right)$, which includes all direct and indirect relations. The equation T = N(*I*−*N*)^−1^ already includes matrix I. I lends the suspicion of repeated calculation. However, the diagonal values of the total relation matrix T may not be all 1, because the principles of the two algorithms inherently differ.

After the adjacency matrix (element is 0 or 1) of ISM is generated, the sum of the adjacency matrix and the identity matrix I performs the power operation of matrix A+ I on a certain integer n until the following equation holds: $A+I\neq {\left(A+I\right)}^{2}\neq {\left(A+I\right)}^{3}\neq \cdots \neq {\left(A+I\right)}^{n}={\left(A+I\right)}^{n+1}=K$. K is the reachability matrix; this power operation is based on Boolean algebra. The elements in the ISM reachability matrix of ISM also fall under the law of transitivity, so the reachability level of each node after passing through paths has a length not greater than (n-1). For graphs with n nodes, the length of the longest path does not exceed (n-1). The total-relation matrix T of DEMATEL does not have this property, but reflects the mutual influence and various levels among different factors and contains sufficient information for a sound analysis. All diagonal elements can be directly changed to 1 in the case discussed here. (The law of transitivity characteristics is discussed further below.)

The lack of scientific standard for setting the threshold value is also problematic. Various threshold-setting techniques have been formulated by experts or decision-makers according to actual problems, but there is no unified scientific standard. Directly setting the threshold to non-0 or 1 actually negates the advantage of DEMATEL’s inclusion of the level of influence between factors. Other threshold determination techniques include the mean method [25], median method [26], μ +σ [27], and μ + 1.5σ [28]. In the present study, the threshold was set via maximum mean de-entropy (MMDE) [29]. Originally a physics concept, entropy describes the state function of system chaos. Informatics researchers later used entropy for information measurement in probability and statistical models.

### MMDE and transitivity check and conversion

To operate MMDE, let a set of random variables X = {*x*_1_,*x*_2_,…,*x*_*n*_} while the relative probability of variables is P = (p_1_, *p*_2_,…,*p*_*n*_}; the information entropy is H(p_1_,p_2_,…p_n_) = -∑*p*_*i*_*ln p*_*i*_, where ∑*p*_*i*_ = 1. If Pi = 0, then *p*_*i*_*ln p*_*i*_ = 0. The de-entropy (H^D^) can be defined as $H^{D}=H(\frac{1}{n},\frac{1}{n},\ldots .,\frac{1}{n})-H(p_{1},p_{2},\ldots ,p_{n})$, which reveals the amount of useful information and reduces informational uncertainty in models such as the DEMATEL-ISM.

The MMDE is implemented in the following steps.

1. Transform the total-relation matrix T into a set of three elements, then arrange the influence values from large to small to form a set T*. The collection consists of {influence level value, row where element is located, column where element is located}.
2. Separately, form the rows (columns) in which all factors in the T* set are located into the T^d^(T^r^) set.
3. Calculate the de-entropy (MDE) values of T^d^ (T^r^) to the i-th element respectively, ${MDE}_{i}^{D}=\frac{H_{i}^{D}}{N(T_{i}^{d})}({MDE}_{i}^{R}=\frac{H_{i}^{D}}{N(T_{i}^{r})})$, where $N(T_{i}^{d})$ is the number of categories of factors in set $T_{i}^{d}(N(T_{i}^{r})$ is the number of categories of factors in set $T_{i}^{r}$).
4. List all sets composed by ${MDE}_{i}^{D}$ and ${MDE}_{i}^{R}$, find the maximum value, then list the rows (columns) larger than ${MDE}_{i}^{D}$ and ${MDE}_{i}^{R}$; delete the repeated rows (columns), leaving only different kinds of elements to form the set.
5. Determine $T_{max}^{d}{(T}_{max}^{d})$: the set established in Step 4 is a three-element set $T_{max}^{d}\;and\;T_{max}^{r}$ with the largest influence value in the corresponding T* set.
6. Calculate T^th^: Take $T_{max}^{d}\cup T_{max}^{r}$ = T^th^, then the minimum influence value in T^th^ set can be used as the threshold value.

It is also important to consider whether the reachability matrix K, which is compared simply with the threshold from matrix H+I, can be directly divided into layers. Some researchers have regarded matrix H as an adjacency matrix rather than a reachability matrix after comparing it with the threshold value. Zhou et al. [30], for example, changed matrix T into an ISM adjacency matrix after comparison with the threshold value and adding I, then obtained a reachability matrix after Boolean algebraic operation. Yu and Shao [31] compared the matrix T with the threshold value (mean value of T), added I to form the ISM adjacency matrix, and then obtained the reachability matrix by Boolean algebra. Weng et al. [32] obtained the reachability matrix after Boolean algebra by first adding I to matrix T, then comparing the threshold value with 0.5 to form the ISM adjacency matrix. It is yet unclear whether it is more appropriate to add I before or after the addition in setting a threshold–re-referencing the adjacency matrix in these cases drives down the overall efficiency of the integration and creates a burdensome calculation process.

Other researchers have directly converted the reachability matrix and then divided it into layers. However, under the law of transitivity of the reachability matrix, the corresponding rows or columns of nodes Si and Sj in the matrix have exactly the same corresponding elements; Si and Sj are on the same loop. The conversion directly from DEMATEL does not necessarily have this property, so an additional transitivity check conversion process is necessary.

**Fig 1:**
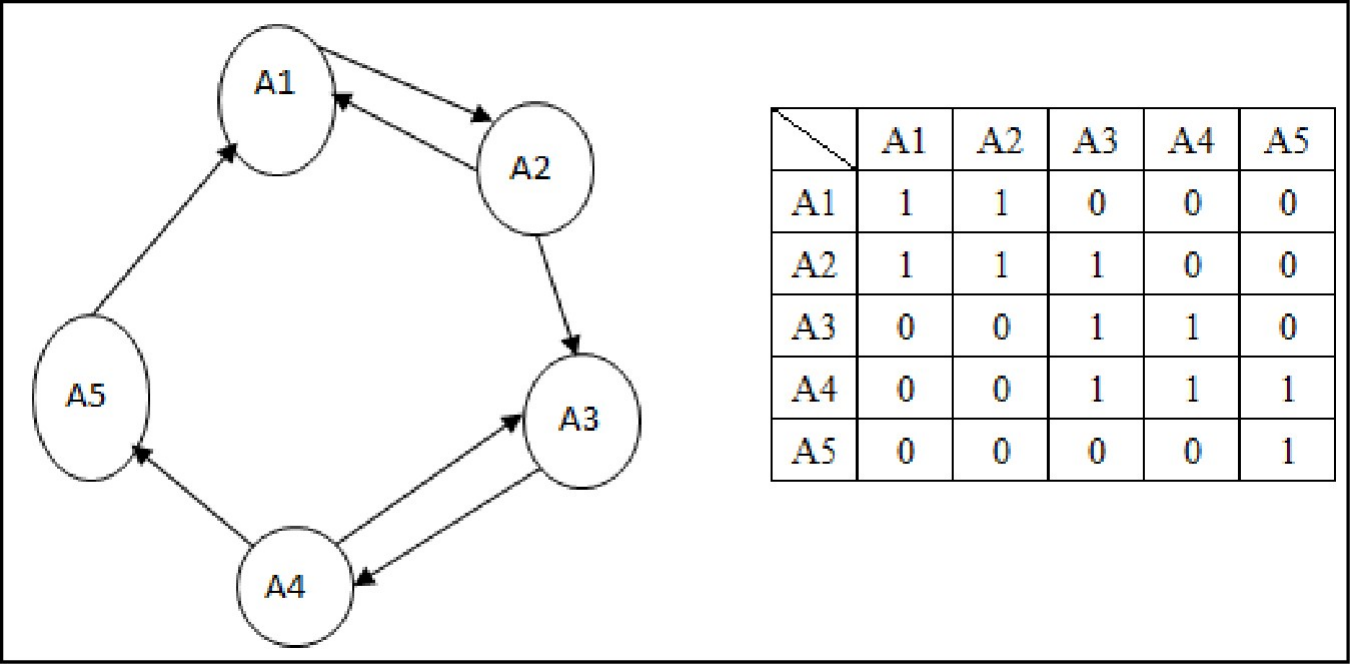
Direct influence relationship and influence relationship matrix of system factors.

To complete the transitivity check, first assume that there are five factors in a certain system. The direct influence relationship between them is shown in the left half of Fig 1 and the direct influence relationship is shown in the right half of Fig 1.

**Fig 2:**
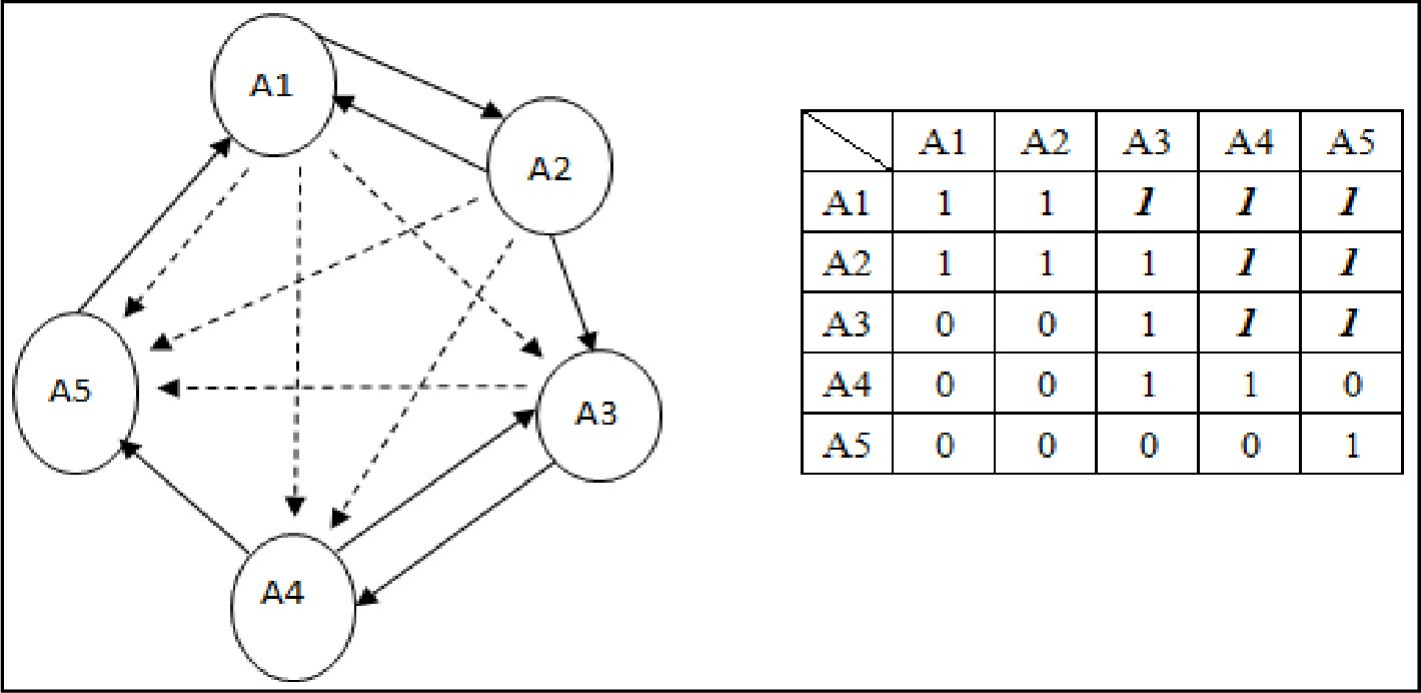
Influence relation and reachability matrix after transitivity check and conversion.

If the matrix is converted from the DEMATEL calculation and compared with the threshold value in Fig 1, it may not be possible to treat it as a reachability matrix. The reachability matrix must include indirect influence and possess law of transitivity characteristics, so if A1 not only directly affects A2 but also affects A3 (because A2 affects A3), then A1 has indirect influence on A3. By analogy, the transitivity of all factors can be checked at this point. If indirect influence is identified, the missing indirect influence relationship between factors should be supplemented via 0 and converted to 1 as marked in bold-italics in the right half of Fig 2.

## Proposed method

The proposed, improved DEAMTEL-ISM integration approach is operated in the following steps.

1. Use DEMATEL to obtain the total-relation matrix T.
2. Calculate the threshold value by MMDE method and transform it into an initial reachability matrix after comparison.
3. Modify/hold at 1 all diagonal elements from the top left to the bottom right of the reachability matrix, then build the final reachability matrix via transitivity check.
4. Finally, divide the reachability matrix into ISM layers.

## Analysis

Previous studies on influence factor selection generally center on health insurance [33–35] and endowment insurance [36]. Chinese social insurance includes five categories: employment injury insurance, endowment insurance, unemployment insurance, maternity insurance, and medical insurance [37]. Issues such as regulations of the township-city floating population on the social security of a given city, and the social environment of places of residence, have significant effects on the social insurance system. Previous research on social integration [38–40] were also referenced for the purposes of this study. A total of 15 influence factors were consolidated as shown in Table 1.

**Table 1:** 15 influence factors.

Coding	Factors
S1	Age
S2	Education level
S3	Payment capacity
S4	Willingness to integrate into the city
S5	Understanding of social insurance
S6	Revenue expectation
S7	Social security demand
S8	Children’s education
S9	Economic pillar
S10	Career stability
S11	Occupational income
S12	Orgaizational system
S13	Cost of living
S14	Difficulty of city integration
S15	Transfer system in different places

### Analysis by improved integration approach

The Guangdong-Hong Kong-Macao Greater Bay Area served as the research area for this study. Seven experts familiar with this field were interviewed. Their interviews were synthesized to establish a direct-relation matrix X, then run through the steps of DEMATEL to secure the total-relation matrix shown in Table 2.

**Table 2:** Direct-relation matrix and total-relation matrix.

*Direct-Relation Matrix*														
0	1	2	0	2	1	2	3	3	0	1	0	0	0	0
0	0	3	2	2	1	2	1	0	2	3	0	0	0	0
0	0	0	1	0	0	0	0	0	0	0	0	0	2	0
0	0	0	0	0	0	1	0	0	0	0	0	0	0	0
0	0	0	0	0	2	0	0	0	0	0	0	0	0	0
0	0	0	0	0	0	0	0	0	0	0	0	0	0	0
0	0	0	0	0	0	0	0	0	0	0	0	0	0	0
0	0	0	3	0	0	3	0	0	0	0	0	0	0	0
0	0	3	2	0	0	0	0	0	0	0	0	0	0	0
0	0	3	0	0	0	0	0	0	0	0	0	0	0	0
0	0	0	2	0	0	0	0	0	0	0	0	0	0	0
0	0	0	0	0	0	0	0	0	0	0	0	0	1	0
0	0	3	2	0	0	0	2	0	0	0	0	0	2	0
0	0	0	3	0	0	0	0	0	0	0	0	0	0	0
0	0	0	0	0	3	0	0	0	0	0	0	0	0	0
*Total-Relation Matrix*														
0	0.06	0.17	0.09	0.13	0.08	0.17	0.19	0.19	0.01	0.07	0	0	0.02	0
0	0	0.21	0.18	0.13	0.08	0.15	0.06	0	0.13	0.19	0	0	0.03	0
0	0	0	0.09	0	0	0.01	0	0	0	0	0	0	0.13	0
0	0	0	0	0	0	0.06	0	0	0	0	0	0	0	0
0	0	0	0	0	0.13	0	0	0	0	0	0	0	0	0
0	0	0	0	0	0	0	0	0	0	0	0	0	0	0
0	0	0	0	0	0	0	0	0	0	0	0	0	0	0
0	0	0	0.19	0	0	0.2	0	0	0	0	0	0	0	0
0	0	0.19	0.14	0	0	0.01	0	0	0	0	0	0	0.02	0
0	0	0.19	0.02	0	0	0	0	0	0	0	0	0	0.02	0
0	0	0	0.13	0	0	0.01	0	0	0	0	0	0	0	0
0	0	0	0.01	0	0	0.0007	0	0	0	0	0	0	0.06	0
0	0	0.19	0.19	0	0	0.04	0.13	0	0	0	0	0	0.15	0
0	0	0	0.19	0	0	0.01	0	0	0	0	0	0	0	0
0	0	0	0	0	0.19	0	0	0	0	0	0	0	0	0

The threshold value was then calculated by MMDE according to the total-relation matrix. The T* set was sorted first. After the calculation steps described above, the maximum MDE^D^ and MDE^R^ were found to be 0.0322 and 0.0446, respectively. The row and column element sets of the largest MDE^D^ and MDE^R^ were combined to determine the minimum influence as 0.063, so the threshold was set to 0.063. The calculation results of each stage are listed in Table 3.

**Table 3:** Threshold results by MMDE.

Item	Data							
The ordered	(0.211,2,3),(0.199,8,7),(0.191,1,8),(0.188,2,11),(0.188,8,4),(0.188,9,3),(0.188,10,3),(0.188,13,3),							
triplets set T*	(0.188,13,4),(0.188,14,4),(0.188,1,9),(0.188,15,6),(0.141,9,4),(0.125,11,4),(0.125,13,8),(0.125,5,6),							
	(0.178,2,4),(0.174,1,7),(0.173,1,3),(0.148,2,7),(0.148,13,14),(0.133,1,5),(0.125,2,5),(0.125,2,10),							
	(0.125,3,14),(0.091,1,4)(0.086,3,4),(0.083,1,6),(0.078,2,6),(0.074,1,11),(0.063,1,2),(0.063,4,7),							
	(0.063,2,8),(0.063,12,14),(0.035,13,7),(0.026,2,14),(0.023,9,14),(0.023,10,14),(0.022,1,14),(0.016,10,4),							
	(0.012,12,4),(0.012,14,7),(0.009,9,7),(0.008,1,10),(0.008,11,7),(0.005,3,7),(0.001,10,7),(0.0007,12,7)							
Dispatch-node set T^d^	{2,8,1,2,8,9,10,13,13,14,1,15,9,11,13,5,2,1,1,2,13,1,2,2,3,1,3,1,2,1,1,4,2,12,13,2,9,10,1,10,12,14,9,1,11,3,10,12}							
Receive-node set T^r^	{3,7,8,11,4,3,3,3,4,4,9,6,4,4,8,6,4,7,3,7,14,5,2,10,14,4,4,6,6,11,2,7,8,14,7,14,14,14,14,4,4,7,7,10,7,7,7,7}							
Value of T^d^ MDE^D^ set	{0,0,0,0.0196,0.0145,0.0141,0.0119,0.0098,0.0081,0.0084,0.0074,0.0065,0.0065,0.0059,0.0091,0.0115,							
	0.0132,0.0148,0.0188,0.0208,0.0192,0.0218,0.0239,0.0265,0.0246,0.0224,0.0244,0.0266,0.0286,0.0308,0.0283,							
	0.0301,0.0285,0.0303,0.0306,0.0322,0.0300,0.0278,0.0294,0.0273,0.0262,0.0277,0.0257,0.0246,0.0235,0.0229}							
Value of T^R^ MDE^R^ set	{0,0,0,0,0,0.0097,0.0268,0.0446,0.0364,0,0303,0.0254,0.0282,0.0239,0.0194,0.0231,0.0280,0.0224,0.0258,							
	0.0240,0.0239,0.0195,0.0196,0.0162,0.0163,0.0136,0.0160,0.0152,0.0155,0.0127,0.0135,0.0137,0.0129,0.0120,							
	0.0137,0.0142,0.0140,0.0144,0.0152,0.0162,0.0168,0.0176,0.0152,0.0162,0.0172,0.0183,0.0195}							
Max MDE^D^	0.0322							
Max MDE^R^	0.0446							
Dispatch-node set of Max. MDE^D^	{2,8,1,2,8,9,10,13,13,14,1,15,9,11,13,5,2,1,1,2,13,1,2,2,3,1,3,1,2,1,1,4,2,12,13}							
	→{2,8,1,9,10,13,14,15,11,5,3,4}							
Receive-node set of Max. MDE^R^	{3,7,8,11,4,3,3,3,4,4,9,6,4,4,8,6,4,7,3,7,14,5,5,10,14,4,4,6,6,11,2,7,8,14}							
	→{3,7,8,11,4,9,6,14,5,10,2}							
T^th^	0.063							

The elements with a total-relation matrix less than the threshold value were changed to 0, those with a total-relation matrix greater than or equal to the threshold value were changed to 1, and the transitivity check was performed after adding the 15-order identity matrix. Many elements showed indirect influence in this case and thus were changed to 1 (bolded in Table 4) to obtain the final reachability matrix.

**Table 4:** Final reachability matrix.

1	1	1	***1***	1	1	1	1	1	***1***	1	0	0	***1***	0
0	1	1	1	1	1	1	1	0	1	1	0	0	***1***	0
0	0	1	1	0	0	***1***	0	0	0	0	0	0	1	0
0	0	0	1	0	0	1	0	0	0	0	0	0	0	0
0	0	0	0	1	1	0	0	0	0	0	0	0	0	0
0	0	0	0	0	1	0	0	0	0	0	0	0	0	0
0	0	0	0	0	0	1	0	0	0	0	0	0	0	0
0	0	0	1	0	0	1	1	0	0	0	0	0	0	0
0	0	1	1	0	0	***1***	0	1	0	0	0	0	1	0
0	0	1	***1***	0	0	***1***	0	0	1	0	0	0	1	0
0	0	0	1	0	0	***1***	0	0	0	1	0	0	0	0
0	0	0	1	0	0	1	0	0	0	0	1	0	1	0
0	0	1	1	0	0	***1***	1	0	0	0	0	1	1	0
0	0	0	1	0	0	***1***	0	0	0	0	0	0	1	0

An ISM hierarchy analysis was performed according to the final reachability matrix; the results are shown in Table 5.

**Table 5:** Hierarchical analysis of proposed approach.

Factors	Reachability set	Antecedent set	R∩A = R	Layer
1	1, 2, 3, 4, 5, 6, 7, 8, 9, 10, 11, 14	1,		
2	2, 3, 4, 5, 6, 7, 8, 10, 11, 14	1, 2		
3	3, 4, 7, 14	1, 2, 3, 9, 10, 13		
4	4, 7	1, 2, 3, 4, 8, 9, 10, 11, 12, 13, 14		
5	5, 6	1, 2, 5		
6	6,	1, 2, 5, 6, 15	√	I
7	7,	1, 2, 3, 4, 7, 8, 9, 10, 11, 12, 13, 14	√	I
8	4, 7, 8	1, 2, 8, 13		
9	3, 4, 7, 9, 14	1, 9		
10	3, 4, 7, 10, 14	1, 2, 10		
11	4, 7, 11	1, 2, 11		
12	4, 7, 12, 14	12,		
13	3, 4, 7, 8, 13, 14	13,		
14	4, 7, 14	1, 2, 3, 9, 10, 12, 13, 14		
15	6, 15	15,		
1	1, 2, 3, 4, 5, 8, 9, 10, 11, 14	1,		
2	2, 3, 4, 5, 8, 10, 11, 14	1, 2		
3	3, 4, 14	1, 2, 3, 9, 10, 13		
4	4,	1, 2, 3, 4, 8, 9, 10, 11, 12, 13, 14	√	II
5	5,	1, 2, 5	√	II
8	4, 8	1, 2, 8, 13		
9	3, 4, 9, 14	1, 9		
10	3, 4, 10, 14	1, 2, 10		
11	4, 11	1, 2, 11		
12	4, 12,14	12,		
13	3, 4, 8, 13, 14	13,		
14	4, 14	1, 2, 3, 9, 10, 12, 13, 14		
15	15,	15,	√	II
1	1, 2, 3, 8, 9, 10, 11, 14	1,		
2	2, 3, 8, 10, 11, 14	1, 2		
3	3, 14	1, 2, 3, 9, 10, 13		
8	8,	1, 2, 8, 13	√	III
9	3, 9, 14	1, 9		
10	3, 10, 14	1, 2, 10		
11	11,	1, 2, 11	√	III
12	12, 14	12,		
13	3, 8, 13, 14	13,		
14	14,	1, 2, 3, 9, 10, 12, 13, 14	√	III
1	1, 2, 3, 9, 10	1,		
2	2, 3, 10	1, 2		
3	3,	1, 2, 3, 9, 10, 13	√	IV
9	3, 9,	1, 9		
10	3, 10	1, 2, 10		
12	12,	12,	√	IV
13	3, 13	13,		
1	1, 2, 9, 10	1,		
2	2, 10	1, 2		
9	9,	1, 9	√	V
10	10,	1, 2, 10	√	V
13	13,	13,	√	V
1	1, 2	1,		VII
2	2,	1, 2	√	VI

### Analysis by traditional integration approach

After adding the 15-order identity matrix to the afore-mentioned total-relation matrix, the threshold value was set to 0.092 according to the consensus reached by the researchers and experts. Elements with a total-relation matrix less than the threshold value were changed to 0 and elements with values greater than or equal to the threshold were changed to 1. The resulting reachability matrix is shown in Table 6.

**Table 6:** Reachability matrix.

1	0	1	0	1	0	1	1	1	0	0	0	0	0	0
0	1	1	1	1	0	1	0	0	1	1	0	0	0	0
0	0	1	0	0	0	0	0	0	0	0	0	0	1	0
0	0	0	1	0	0	0	0	0	0	0	0	0	0	0
0	0	0	0	1	1	0	0	0	0	0	0	0	0	0
0	0	0	0	0	1	0	0	0	0	0	0	0	0	0
0	0	0	0	0	0	1	0	0	0	0	0	0	0	0
0	0	0	1	0	0	1	1	0	0	0	0	0	0	0
0	0	1	1	0	0	0	0	1	0	0	0	0	0	0
0	0	1	0	0	0	0	0	0	1	0	0	0	0	0
0	0	0	1	0	0	0	0	0	0	1	0	0	0	0
0	0	0	0	0	0	0	0	0	0	0	1	0	0	0
0	0	1	1	0	0	0	1	0	0	0	0	1	1	0
0	0	0	1	0	0	0	0	0	0	0	0	0	1	0
0	0	0	0	0	1	0	0	0	0	0	0	0	0	1

A factor hierarchy analysis was then performed via ISM in each step of the calculation process. The results are shown in Table 7.

**Table 7:** Hierarchical analysis of traditional approach.

Factors	Reachability set	Antecedent set	R∩A = R	Layer
1	1, 3, 5, 7, 8, 9	1,		
2	2, 3, 4, 5, 7, 10, 11	2,		
3	3, 14	1, 2, 3, 9, 10, 13		
4	4,	2, 4, 8, 9, 11, 13, 14	√	I
5	5, 6	1, 2, 5		
6	6,	5, 6, 15	√	I
7	7,	1, 2, 7, 8	√	I
8	4, 7, 8	1, 8, 13		
9	3, 4, 9	1, 9		
10	3, 10	2, 10		
11	4, 11	2, 11		
12	12,	12,	√	I
13	3, 4, 8, 13, 14	13,		
14	4, 14	3, 13, 14		
15	6, 15	15,		
1	1, 3, 5, 8, 9	1,		
2	2, 3, 5, 10, 11	2,		
3	3, 14	1, 2, 3, 9, 10, 13		
5	5,	1, 2, 5	√	II
8	8,	1, 8, 13	√	II
9	3, 9	1, 9		
10	3, 10	2, 10		
11	11,	2, 11	√	II
13	3, 8, 13, 14	13,		
14	14,	3, 13, 14	√	II
15	15,	15,		
1	1, 3, 9	1,		
2	2, 3, 10	2,		
3	3,	1, 2, 3, 9, 10, 13	√	III
9	3, 9	1, 9		
10	3, 10	2, 10		
13	3, 13	13,		
15	15,	15,	√	III
1	1, 9	1,		
2	2, 10	2,		
9	9,	1, 9	√	IV
10	10,	2, 10	√	IV
13	13,	13,	√	IV
1	1,	1,	√	V
2	2,	2,	√	V

## Comparison and results

The influence factors can be divided into seven layers based on the proposed integration approach: the first layer is S1 and S6, the second layer is S4, S5, and S15, the third layer is S8, S11, and S14, the fourth layer is S3 and S12, the fifth layer is S9, S10, and S13, the sixth layer is S2, and the seventh layer is S1. The results obtained by traditional integration approach are S1, S6, S7, and S12 as the first layer, S5, S8, S11, and S14 as the second, S3 and S15 as the third, S9, S10, and S13 as the fourth, and S1 and S2 as the fifth. These are substantial differences on the whole. S5, S6, and S7 are similar while S3, S4, S8, S9, S10, S11, S13, S14, and S15 differ only slightly, but the respective approaches produce S1 and S2 as factors of a different order. The difference between S12 is especially large–the factor of the S12 unit system, logically speaking, should not be a first-order factor. This indicates that the traditional integration approach may not be accurate. The hierarchical structure analysis results of the two approaches are shown in Table 8.

**Table 8:** Hierarchical structure analysis results of two approaches.

Layer	L1	L2	L3	L4	L5	L6	L7
Novel approach	6,7	4,5,15	8,11,14	3,12	9,10,13	2	1
Traditional approach	4,6,7,12	5,8,11,14	3,15	9,10,13	1,2		

**Fig 3:**
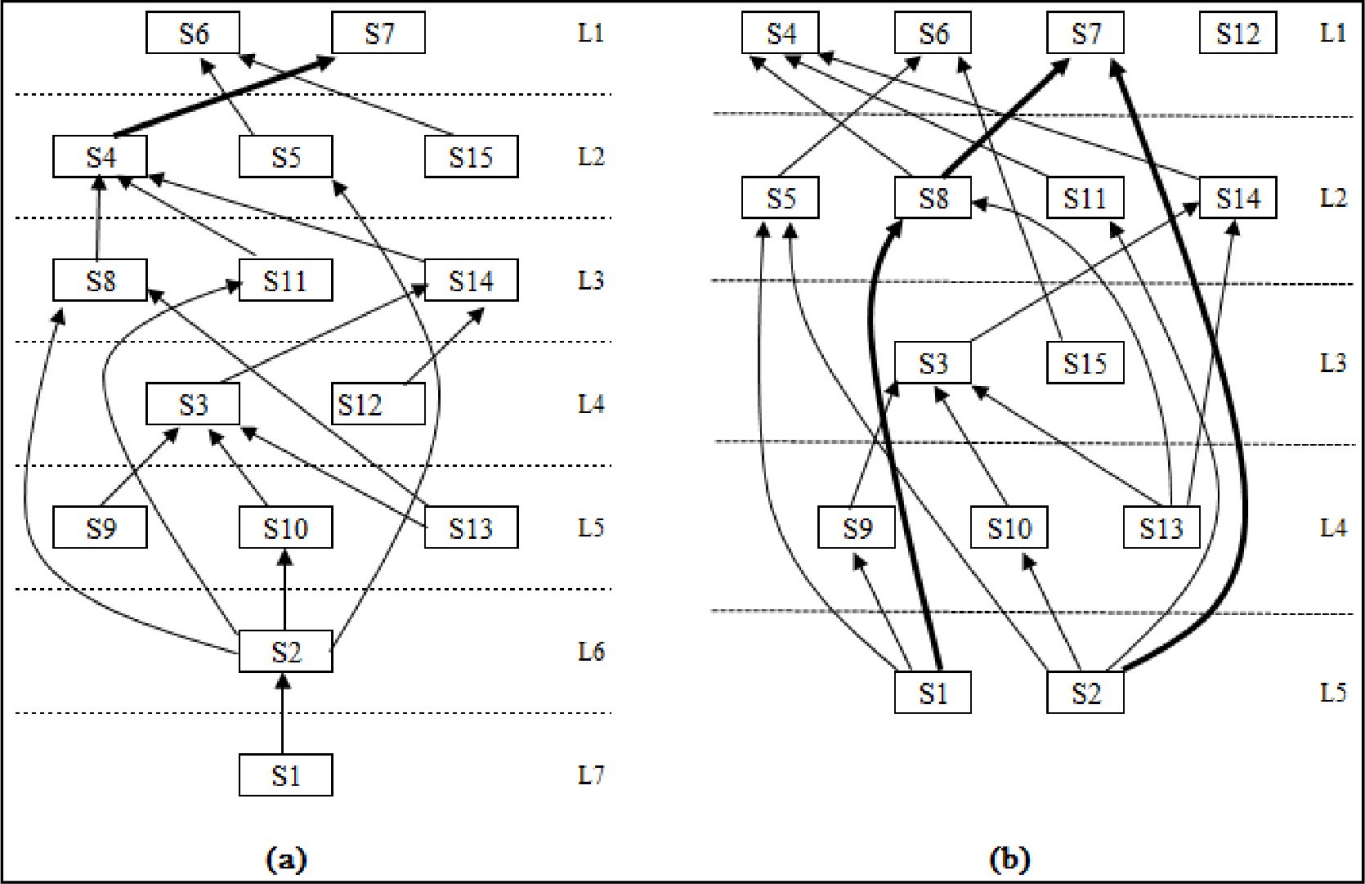
Factor layers and inner influence relationships of two approaches.

An inner interaction diagram of the influence factors makes the differences between the two approaches even more stark. Fig 3(A) shows the factor layer and inner influence relationship diagram obtained by the proposed approach, where S4 directly affects S7. Fig 3(B) shows the factor layer and inner influence relationship diagram obtained by the traditional integration approach, where S1 directly affects S8 and S4 does not affect S7; S2 directly affects S7 and S8 directly affects S7. The proposed approach shows an indirect influence relationship among these three factors. The above differences are marked in the figure by thick lines. It appears that the traditional integration approach does not necessarily provide more accurate results in terms of the hierarchical factor structure and the inner relationships among influence factors.

## Conclusion

Integrating DEMATEL and ISM is an interesting analysis technique, as it may lend simplicity while minimizing the computational burden of the two respective methods. However, the traditional approach to integration has significant drawbacks–mainly in terms of the threshold determination and transitivity checking process. The system structure obtained by this traditional integration method may not completely conform to the strict definition of “hierarchical”, so it is not necessarily possible to accurately determine the hierarchical structure of factors and the inner relationships among them. This can lead to deviation in the ISM analysis results and drive down the effectiveness of any countermeasures implemented.

In this study, an improved DEMATEL-ISM integration approach was established which includes an MMDE approach to determine the threshold, and composition of a transitivity check correction reachability matrix. The influence factors of the social insurance willingness of the rural-urban floating population in the Guangdong-Hong Kong-Macao Greater Bay Area was taken as an example to test the proposed improved integration approach by comparison against the traditional integration method. Differences emerged between the two approaches, including the hierarchical structure and inner influence relationships, which were not extreme but do still merit careful consideration. This comparative analysis also confirmed the feasibility and effectiveness of the improved integration approach. The results of this work may serve as a helpful reference for further research on this topic.

## Supporting information

S1 Dataset (XLSX)
